# Left Ventricular Diastolic Function Following Anthracycline-Based Chemotherapy in Patients with Breast Cancer without Previous Cardiac Disease—A Meta-Analysis

**DOI:** 10.3390/jcm10173890

**Published:** 2021-08-29

**Authors:** Raluca I. Mincu, Lena F. Lampe, Amir A. Mahabadi, Rainer Kimmig, Tienush Rassaf, Matthias Totzeck

**Affiliations:** 1 West German Heart and Vascular Center, Department of Cardiology and Vascular Medicine, University Hospital Essen, Hufelandstr. 55, 45147 Essen, Germany; Raluca-Ileana.Mincu@uk-essen.de (R.I.M.); Lena.Lampe@stud.uni-due.de (L.F.L.); Amir-Abbas.Mahabadi@uk-essen.de (A.A.M.); Tienush.Rassaf@uk-essen.de (T.R.); 2 Clinic for Obstetrics and Gynecology, Essen University Hospital, 45147 Essen, Germany; Rainer.Kimmig@uk-essen.de

**Keywords:** diastolic dysfunction, breast cancer, echocardiography, anthracycline, cardiotoxicity, diagnosis and prevention

## Abstract

Background: Anthracycline-based chemotherapy (ANT) remains among the most effective therapies for breast cancer. Cardiotoxicity from ANT represents a severe adverse event and may predominantly manifest as heart failure. While it is well-recognised that left ventricular systolic heart failure assessment is key in ANT-treated patients, less is known about the relevance of LV diastolic functional impairment and its characterisation. Methods: Studies reporting on echocardiographic diastolic function parameters before and after ANT in breast cancer patients without cardiac disease were included. We evaluated pulsed wave (E/A ratio and mitral E-wave deceleration time (EDT)) and tissue Doppler (mean velocities of the mitral ring in the early diastole (e′) and E/e′ ratio) echocardiographic parameters. Results: A total of 892 patients from 13 studies were included. E/A ratio was significantly reduced at the end of ANT while EDT was not influenced by ANT. Additionally, e’ and E/e’ ratio showed no significant change after ANT. A modest reduction in LV ejection fraction and global longitudinal strain was observed at the end of ANT therapy. Conclusions: ANT had a modest early impact on E/A ratio, without changing EDT, e’, or E/e’ in patients with breast cancer without cardiac disease. Randomised studies on larger populations, using new parameters are required to define the role of diastolic dysfunction in the early diagnosis of ANT-induced cardiotoxicity.

## 1. Introduction

Breast cancer accounts for 30% of all new cancer diagnoses in women across all ages [1]. The therapy depends on the molecular cancer subtype, tumor stage, and risk of relapse and consists of a combination of surgery, chemotherapy, targeted and immune therapy, radiotherapy, and endocrine therapy. Anthracylines and taxanes are conventional chemotherapies for HER2 positive and triple negative breast cancer subtypes and remain a cornerstone of breast cancer therapy, considering the reduction of 10-year mortality by about one third [2,3,4].

The most severe adverse event of anthracycline-based chemotherapy (ANT) is cardiotoxicity. The main mechanisms of ANT related cardiotoxicity are the production of free radicals and reactive oxygen species [5] and the inhibition of topoisomerase IIβ through disruption in the function of deoxyribonucleic acid, with consecutive damage of multiple cardiomyocyte components and the activation of cell death pathways [6,7,8].

The poor prognosis and the lack of specific treatment options for anthracycline-induced cardiotoxicity impose extensive efforts for the early detection of cardiac dysfunction during ANT, in order to avoid progression to heart failure [9]. After initiating ANT, the focus of the follow-up examinations should be on the early detection of cardiotoxicity, defined according to the current guidelines as a reduction in left ventricular ejection fraction (LVEF) of more than 10% points to a value below the lower limit of normal (50%) or a relative procentual reduction of global longitudinal strain (GLS) of more than 15% compared to the baseline value [10]. Changes in LVEF and GLS after cancer therapy represent standard diagnostic tools for cardiotoxicity, with changes in GLS occurring earlier compared to changes in LVEF [11]. Although the development of diastolic dysfunction could precede the changes in LVEF, the presence of diastolic dysfunction is not included in the definition of cardiotoxicity [10,12,13]. However, data regarding the superiority of GLS over the diastolic dysfunction in detecting the left ventricular (LV) dysfunction are conflicting [11,14,15,16]. Aditionally, diastolic dysfunction was associated with subsequent heart failure and higher mortality in population-based cohorts [17,18,19] and plays a fundamental role in the diagnosis of heart failure with preserved ejection fraction [20,21]. 

Left ventricular (LV) diastolic dysfunction represents the inability of LV to fill to a normal end-diastolic volume and to provide an adequate stroke volume [22,23]. LV diastolic dysfunction is a result of impaired LV relaxation and increased LV stiffness, with consequently increased LV filling pressures. Echocardiography is the primary imaging modality used to diagnose LV diastolic dysfunction. Echocardiographic parameters of diastolic function derive from pulsed wave Doppler interrogation of the mitral valve (MV) inflow (E/A ratio, MV E-wave deceleration time (EDT)) or from tissue Doppler velocities (e’ and E/e’ ratio). E/A ratio is defined as MV E-wave velocity divided by A-wave velocity, while EDT represents the time from peak E-wave to zero-velocity baseline. The e’ defines the mean velocities at the mitral ring level and E/e’ ratio is calculated as ratio between MV E-wave velocity divided by mitral annular e’ velocity. E/A and EDT are used to determine the LV filling pattern: normal, impaired relaxation, pseudnormal or restrictive, while e’ correlates with LV relaxation and LV stiffness, and the E/e’ ratio is used to determine LV filling pressures. The assessment of diastolic function through echocardiography is challenging and requires an integrative assessment of many separate parameters, including the parameters described above, together with left atrial volume and tricuspid valve regurgitation velocities, according to the latest guidelines of diastolic function assessment [24].

Most of the studies on patients with breast cancer treated with ANT describe a reduction in LV systolic function using LVEF or GLS, while diastolic dysfunction has been reported as a secondary endpoint, using separate echocardiographic parameters of diastolic dysfunction in non-randomised studies on a small number of subjects [25,26,27,28,29]. Randomised studies on the efficiency of classical heart failure therapies as cardio-protective treatments during chemotherapy have reported diastolic function after ANT on a small number of subjects [28,30,31]. Recently, one of the most comprehensible studies regarding diastolic function in patients with breast cancer treated with ANT and trastuzumab published data on 361 patients and showed a modest decrease of the diastolic function in the long-time follow-up [32]. Studies on small cohorts of patients with breast cancer on ANT showed isolated reductions of LV diastolic function parameters [26,29,33,34]. 

Consequently, available data regarding the impact of ANT on diastolic function is scarce and conflicting. This analysis aims to determine if an alteration in E/A, DTE, e’ or E/e’, as the most frequent reported echocardiographic parameters of diastolic dysfunction, could be used to early detect and to predict ANT related cardiotoxicity in patients with breast cancer without cardiac disease. 

## 2. Materials and Methods

This meta-analysis was performed in accordance with the PRISMA (Preferred Reporting of Items for Systematic Meta-Analysis) guidelines and followed the Cochrane Handbook for Systematic Reviews of Interventions recommendations [35,36]. The study was registered with PROSPERO (CRD42021221893).

### 2.1. Sources of Information and Search Strategies

A systematic search was conducted through Pubmed, Cochrane, Embase, and Web of Science databases using MeSH (Medical Subject Headings) terms and free text to identify relevant studies published in English until 31st of October 2020, according to the inclusion and exclusion criteria.

Only those studies that complied with all the inclusion criteria listed below were included:

1. Prospective or retrospective, randomised or non-randomised studies reporting on diastolic function parameter assessed through echocardiography in patients with breast cancer before and after ANT.
2. Follow-up time more than 6 weeks
3. Sample size > 20 patients

All the studies that fulfilled one or more of the criteria listed below were excluded from the meta-analysis:

1. Abstracts, reviews, animal and in vitro studies, meta-analyses, and case-reports.
2. Studies on patients with other cancer types except breast cancer (e.g., hematologic cancers) treated with ANT.
3. Studies including patients with established cardiovascular disease.
4. Studies that did not report the selected outcomes.
5. Subgroup population studies: elderly population, pediatric population.

After removing duplicates, RM and LL independently reviewed the abstracts. Any discrepancies in results between the two investigators were resolved by discussion with MT. When the inclusion criteria appeared to be met, the entire text was reviewed. At the end of the review process, the full texts of the studies considered eligible were reviewed by all investigators.

### 2.2. Data Extraction and Quality Assessment

Two authors (RM and LL) independently performed the data extraction using a standard data extraction form that contained the following fields: publication details (name of the first author and year of publication); study design; characteristics of the study population (breast cancer type, sample size, age); cumulative dose of anthracyclines; follow-up timing; diastolic function parameters; and the main findings of each study.

The trial quality was assessed using the Newcastle Ottawa Scale [37], considering that the Cochrane Handbook risk of bias refers especially to randomised trials. According to this scale, each study is judged on: (1) selection of the exposed cohort, (2) comparability of cohorts, and (3) assessment of outcomes. A score consisting of up to nine stars is awarded to each study. 

### 2.3. Study Endpoints

The parameters of diastolic function were evaluated through echocardiography at two different timepoints: at baseline (before ANT start) and follow-up (after the last cycle of ANT). When a follow-up visit after the last cycle ANT was not reported, we used data reported at the six-month follow-up evaluation.

The primary study endpoints consisted of changes of the following diastolic function parameters:

(1) E/A ratio calculated as the ratio between mitral early E-wave filling velocity (E) and mitral atrial A-wave filling velocity (A) using pulsed Doppler interrogation at the level of the mitral valve;
(2) mitral E-wave deceleration time (EDT) calculated as the time between the peak E-wave velocity to zero-velocity baseline;
(3) mean velocities of the mitral ring in the early diastole (e’) assessed as the mean between the tissue Doppler mitral annular lateral and median velocities;
(4) E/e’ ratio calculated as ratio between E and e’.

As secondary endpoints we analysed changes in LVEF and GLS between the two assessment timepoints. Additionally, we analysed the influence of high doses ANT and the impact of cardioprotective therapy on the LV diastolic function.

### 2.4. Statistical Analysis

The meta-analysis included patients with breast cancer without cardiac disease treated with ANT. The parameters of diastolic function were assessed at two different timepoints: baseline and follow-up. The mean values and standard deviations of the E/A, EDT, e’, and E/e’ at baseline were compared with the mean values at follow-up in the same population study. The data are expressed as difference in means (DM) and 95% confidence intervals (CI) and for each parameter. A positive value of DM represents a reduction of the mean value of E/A, EDT, e’, and E/e’ at the follow-up, while a negative value of DM represents an increase in the mean value of E/A, EDT, e’, and E/e’ at follow-up. We used random-effects models for all analyses. Heterogeneity between studies was tested using Q statistics, and inconsistencies were determined using the *I*^2^ statistical test. We considered the presence of significant heterogeneity at a 10% level of significance. A value of *I*^2^ of 0–40% denotes that heterogeneity might not be important, 30–60% may represent moderate heterogeneity, 50–90% may represent substantial heterogeneity, and 75–100% may represent considerable heterogeneity [35]. The presence of publication bias was assessed using the funnel plot test (Egger’s test). Studies with high precision are plotted near the average, and studies with low precision are spread evenly on both sides of the average, creating a roughly funnel-shaped distribution. Deviation from this shape indicates publication bias [38]. The funnel plot test was not used when the analysis included less than 10 studies [35]. All analyses were conducted using the comprehensive meta-analysis software (Biostat Inc., Englewood, NJ, USA).

## 3. Results

### 3.1. Study Selection

The meta-analysis included 13 studies with a total of 892 patients with breast cancer that received ANT [25,26,27,28,29,30,31,32,33,34,39,40,41]. The PRISMA selection flowchart is depicted in Supplementary Figure S1. Details about study population, mean age in each group, doses of ANT, reported echocardiographic diastolic function parameters, main findings of each included study, and data about the main predictors of cardiotoxicity for each study are listed in Table 1. Metastasis rate was reported in two studies with values of 0.5% and 15%, respectively, as reported in only two included studies [30,32]. Studies were excluded because the published data format could either not be analyzed [42,43], or they studied the diastolic function in a mixed cancer population [44,45], or they provided no data regarding diastolic function [46,47], or because they published data on specific subgroups [48,49,50]. 

### 3.2. Primary Endpoints

#### E/A Ratio

**Figure 1:**
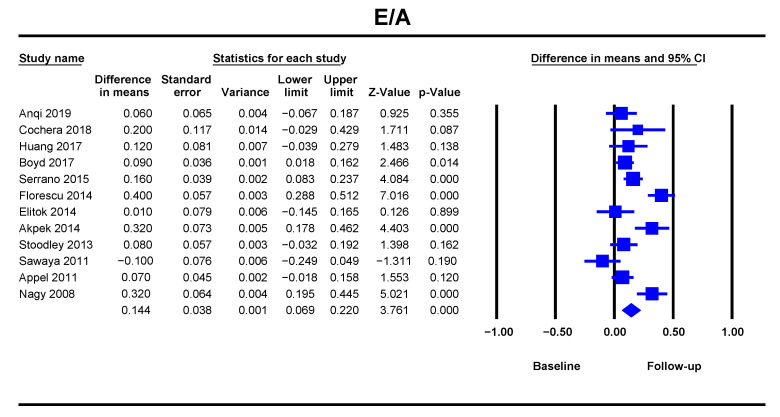
Overall and individual study estimates of the difference in means (DM) for E/A ratio before and after anthracycline-based chemotherapy. Square boxes denote DM for each study, parallelogram boxes denote the overall DM, and horizontal lines represent 95% confidence intervals. E/A = mitral E-wave filling velocity/mitral A-wave filling velocity.

The E/A ratio was significantly decreased at follow-up with a DM of 0.14, 95% CI [0.06–0.22], *p* < 0.001 (Figure 1). The analysis included twelve studies with 673 patients, and showed a low heterogeneity with an I^2^ value of 22.28%. The risk of bias was significant (Supplementary Figure S2). The difference in means suggests a tendency for impaired relaxation.

#### EDT

**Figure 2:**
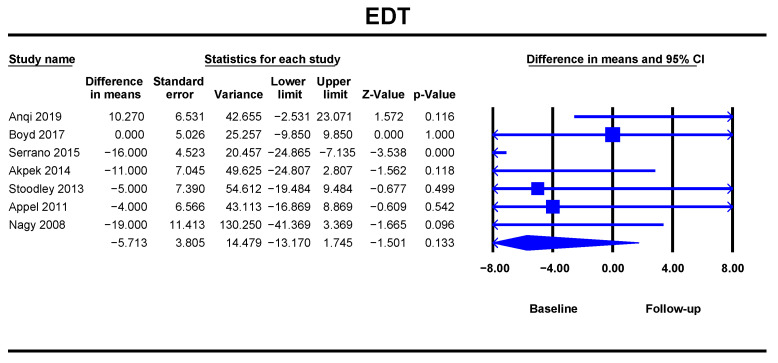
Overall and individual study estimates of difference in means (DM) for the E wave deceleration time before and after anthracycline-based chemotherapy. Square boxes denote DM for each study, parallelogram boxes denote the overall DM, and horizontal lines represent 95% confidence intervals. EDT = E wave deceleration time.

ANT led to a statistically insignificant increase in EDT (DM = −5.71, 95% CI [−13.17–1.74], *p* = 0.133) at follow-up (Figure 2). The analysis included seven studies with 477 breast cancer patients and showed a low heterogeneity with an I^2^ value of 2.89%.

#### e’

**Figure 3:**
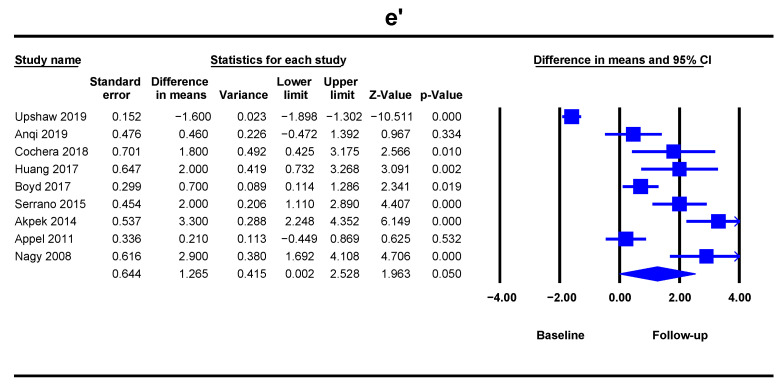
Overall and individual study estimates of difference in means (DM) for the e’ mean velocity of the mitral ring before and after anthracycline-based chemotherapy. Square boxes denote DM for each study, parallelogram boxes denote the overall DM, and horizontal lines represent 95% confidence intervals.

The e’ decreased at follow-up, but did not reach the statistical significance (DM = 1.26, 95% CI [0.002–2.52], *p* = 0.050) when studied on 717 patients from nine studies (Figure 3). The heterogeneity was low.

#### E/e’

**Figure 4:**
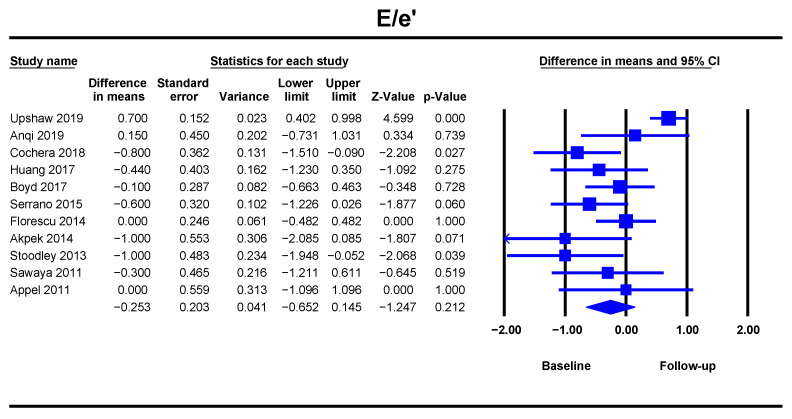
Overall and individual study estimates of difference in means (DM) for the E/e’ ratio before and after anthracycline-based chemotherapy. Square boxes denote DM for each study, parallelogram boxes denote the overall DM, and horizontal lines represent 95% confidence intervals. E/e’ ratio = mitral E-wave filling velocity/ mean velocities of the mitral ring in the early diastole.

The analysis of 812 breast cancer patients from eleven studies showed no influence of ANT of the E/e’ (DM = −0.25, 95%CI [−0.65–0.14], *p* = 0.212) (Figure 4). Heterogeneity was high with an I^2^ value of 72.6%, and the risk of bias was significant (Supplementary Figure S3).

### 3.3. Secondary Endpoints

#### LVEF

**Figure 5:**
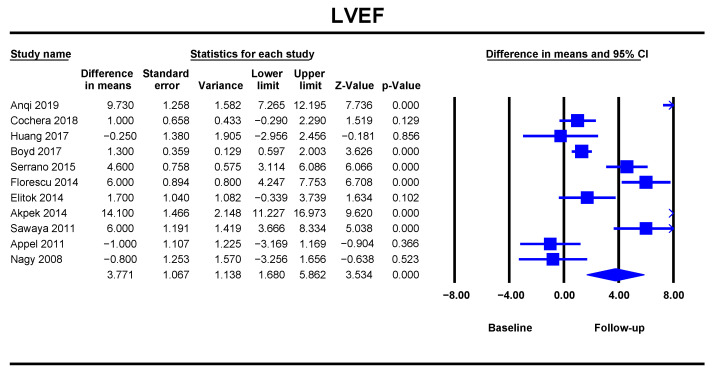
Overall and individual study estimates of difference in means (DM) for the left ventricular ejection fraction (LVEF) before and after anthracycline-based chemotherapy. Square boxes denote DM for each study; parallelogram boxes denote the overall DM, and horizontal lines represent 95% confidence intervals.

When analysing the changes in LVEF at baseline and follow-up, a significant decrease in LVEF could be demonstrated at the end of ANT (DM = 3.77, 95%CI [1.68–5.86], *p* < 0.001). The data was obtained from eleven studies with 621 patients (Figure 5). The heterogeneity was low, and the risk of bias was significant (Supplementary Figure S4).

#### GLS

**Figure 6:**
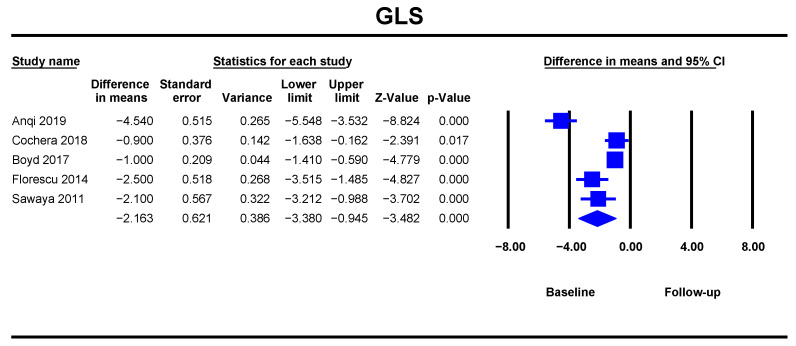
Overall and individual study estimates of difference in means (DM) for global longitudinal strain (GLS) before and after anthracycline-based chemotherapy. Square boxes denote DM for each study; parallelogram boxes denote the overall DM, and horizontal lines represent 95% confidence intervals.

ANT leads to a reduction in GLS at follow-up (DM = −2.16%, 95%CI [−3.38–−0.94], *p* < 0.001). Data was analysed from five studies with 293 patients and showed low heterogeneity (Figure 6).

#### High-Dose ANT

220 patients receiving ANT dose over 400 mg/m^2^ doxorubicin or epirubicin therapeutic equivalent from three studies [29,32,40] showed a significant change in E/A and e’ at follow-up (DM = 0.26, 95%CI [0.07–0.45], *p* = 0.007 and DM = 1.06, 95%CI [0.05–2.08], *p* = 0.040 respectively). The DTE and E/e’ were similar between the two timepoints.

## 4. Discussion

This meta-analysis studied the influence of ANT on the LV diastolic function in 892 patients with breast cancer from 13 studies. The main findings of the study were (a) The E/A ratio showed a modest decrease at the follow-up timepoint; (b) EDT was not influenced by ANT; (c) Neither the early diastolic e’ velocity of the mitral ring, nor the E/e’ ratio was significantly alterated at follow-up; (d) LVEF and GLS showed a significant reduction at the end of the therapy; (e) Patients receiving ANT doses over 400 mg/m^2^ had a significant change in E/A and e’ at follow-up. 

The current data could neither support the use of these parameters for the early diagnosis of ANT cardiotoxicity, nor their use as predictors of ANT cardiotoxicity. The results demonstrate a modest reduction in E/A ratio after ANT, with no influence on EDT, e’ or E/e’ at the end of ANT. Whether ANT induces an alteration of LV diastolic function is incompletely studied. Echocardiographic diastolic function parameters depend on cardiac preload, cardiac rhythm, age, and differences in measurement technique. In cancer patients preload could be easily influenced by dehidration because of gastrointestinal adverse events during chemotherapy. This may explain the observed changes in E/A after therapy [51]. The loading conditions would also influence E/e’ ratio. Additionally, sinus tachycardia is common in patients unter chemotherapy, which determines a false reduction of E/A because of fusion of E and A waves. Additionally, E/A decreases with age and differences in measurement technique between different laboratories could also influence the results [24]. 

The assessment of the diastolic function after ANT has been performed on a small number of patients with short follow-up time. Recently, persistent worsening of diastolic function in 361 patients with breast cancer detected through early reductions in E/A and e’ and increase in E/e’ over a median follow-up time of 2.1 years was reported [32]. However, these results could not be correlated with the occurrence of cardiotoxicity in the affected patients. The second large study on the diastolic function after ANT could show early reduction in the E/A ratio and e’ on 140 patients [39], while other studies on 85 and 80 patients with breast cancer showed a decrease in E/A and e’ and an increase in EDT and E/e’ or only a reduction of E/A ratio, respectively [25,41]. Studies on small cohorts of patients with breast cancer on ANT showed isolated reductions of echocardiographic parameters of diastolic function of LV [26,29,33,34]. 

The demonstrated reduction in LVEF, although statistically significant, was actually modest when evaluating the difference in means at the end of ANT. The current European Society of Cardiology position paper on cancer treatments and cardiovascular toxicity define LVEF and GLS as parameters to diagnose cardiotoxicity, while parameters of diastolic function do not belong to the standard [10]. The use of LVEF to detect cardiotoxicity remains a matter of debate [11]. The myocardium has the ability to recruit new contractile myocytes during ANT. Consequently, a reduction of LVEF occurs in late stages of myocardial disease. Aditionally, the measurement of LVEF is load-dependent, changes in load being frequent in cancer patients, due to gastro-intestinal adverse events during therapy [52]. Considering the fact that a reduction in GLS was a secondary endpoint in this analysis, we included here only the studies reporting on both GLS and diastolic dysfunction at the end of therapy. The interpretation of an impairment in GLS should consider other important studies reporting on GLS after ANT, which was beyond our scope. It has been demonstrated that an impairment of GLS precedes a reduction in LVEF and that a relative reduction of 15% of GLS predicts a reduction in LVEF in the early stages of cancer-therapy [11,53]. GLS measurement demonstrated superiority over diastolic dysfunction in patients with diabetes mellitus and preserved ejection fraction [15,54], which could not be confirmed in other studies [14]. Studies showed an association of diastolic function after ANT with a modest reduction in LVEF and GLS [32], demonstated a predictive value of e’ velocity for a GLS reduction [39], or highlighted the increased specificity of diastolic dysfunction through tissue Doppler echocardiography over classical parameter [34], while the predictive value of GLS for the development of cardiotoxicity has been constantly demonstrated [26,27,29,33]. Additionally, systolic longitudinal rotation and diastolic strain rate were identified as early diagnostic tools to detect cardiotoxicity [39,40]. 

High-dose ANT caused an alteration of both E/A and e’, explained by the impairment of LV relaxation [55]. 

When analysing the control groups of the cardioprotection studies, the results vary from significant changes in tissue Doppler e’ and E/e’ parameter, to no changes in the echocardiographic parameters of diastolic function [28,30,31]. Additionally, cardioprotective therapy showed a limited efficiency in preventing cardiotoxicity in larger analyses [56].

The occurrence of diastolic dysfunction after ANT in patients with breast cancer without cardiac disease remains an open question for the clinical practice. Avilable data show conflicting results due to the lack of standardisation of the echocardiographic parameters of diastolic function, together with the lack of prognostic significance of the changes of these parameter. Consequently, a robust conclusion regarding the diastolic function after ANT is at this timepoint impossible. When assessing the diastolic function in patients with cancer, new echocardiographic parameter should be taken into consideration. Our data included two studies that reported on early diastolic strain rate as a parameter of diastolic function, yet with contradictory results [27,40]. 

Cardio-oncology units are essential for the assessment of cardiovascular function, including the standardised evaluation of diastolic function in cancer patients [57,58,59]. Through the serial and standardised evaluation of diastolic function in cardio-oncology units, together with the use of novel parameters e.g. left atrial function parameter, speckle tracking strain analysis or multimodality imaging techniques, evidence regarding the importance of diastolic dysfunction in the management of oncological patients should be generated. 

### Limitations

This analysis has some limitations that should be addressed. Firstly, most of the studies included in the analysis are not-randomised prospective cohorts, with increases in the risk of bias. Secondly, the analysis could not make a statement regarding the grade of diastolic dysfunction in the entire group of patients, because this requires an integrative assessment of more echocardiographic diastolic function parameters. Thirdly, a correlation with a pathological change in the described parameter with the appearance of ANT related adverse events could not be established. Fourthly, the lack of standardisation for the reporting of echocardiographic parameters to define the diastolic dysfunction could alter the general results.

## 5. Conclusions

The assessment of LV diastolic function after ANT in patients with breast cancer without cardiovascular disease using echocardiography showed a modest early change in E/A ratio, without changes in EDT, e’, or E/e’. Other parameters should be considered to determine the diagnostic and prognostic role of LV diastolic function in ANT-related cardiotoxicity.

## Figures and Tables

**Table 1:** Studies reporting on diastolic function evaluated through echocardiography in patients treated with anthracycline-based chemotherapy.

First Author and Publication Year	Study Design	Cancer Entity	Metastatic Disease (%)	Sample Size (Number of Patients)	Mean Age	CV Risk Factors	Cumulative Dose of Anthracycline	Follow-Up Timing	Diastolic Function Parameter	Impact on the Diastolic Function	Cardiotoxicity Predictors
Upshaw 2019 [32]	Longitudinal cohort	Breast cancer	0.5%	219	49.0 (41.0–57.0)	Arterial hypertension 32% Diabetes mellitus 9% Hyperlipidemia 21% Tobacco use 7%	Doxorubicin 240 mg/m^2^	Baseline and 6, 12, 24, 36 months after therapy.	E/A, E/e’, LA volume index, deceleration time, septal e’, lateral E’, TR velocity, IVRT, diastolic dysfunction grade	Early occurrence of reductions in the E/A ratio and e’ and increases in the E/e’ ratio and persistence over the long term	Abnormal or worsening of diastolic function with therapy is associated with a small increased risk of LVEF reduction, worse longitudinal strain, and an increased risk of cancer therapeutics-related cardiac dysfunction.
Anqi 2019 [33]	Longitudinal cohort	Breast cancer	NR	40	47.27 ± 9.9	Arterial hypertension 25%	Anthracyclines 363.90 ± 107.77 mg/m^2^	Baseline and after each of the 6 cycles	E/A, DT, LA volume index, E/e’, e’, diastolic strain by speckle tracking	LVEF reduction, global longitudinal strain reduction, E’ reduction	LVEF and GLS decreased gradually with higher doses of ANT.
Cochera 2018 [31]	Longitudinal cohort	Breast cancer	NR	30 without nebivolol	52 ± 11	Arterial hypertension 13% Tobacco use 6%	Doxorubicin 520 ± 8 mg/m^2^	Baseline, after 6th cycle of Chemotherapy	E, A, E/A, IVRT, e’ lateral, e’ septal, strain by speckle tracking	Significant changes in e’ septal, e’ lateral, E/e’, LVEF, longitudinal strain, and strain rate.	Longitudinal and radial ventricular strain and could identify patients at risk of developing irreversible cardiotoxicity under anthracycline therapy.
Huang 2017 [40]	Longitudinal cohort	Breast cancer	NR	43	49 ± 8	Arterial hypertension 0% Diabetes mellitus 0% Hyperlipidemia 0% Tobacco use 0%	Epirubicin 524 ± 141 mg/m^2^ Therarubicin 336 ± 115 mg/m^2^	Baseline, 3 weeks, 6 months	E/A, DT; pulmonary vein, e‘, A‘, E/A‘, LA volume, diastolic Strain	e‘ septal decrease, reduction of peak early-diastole LV wall velocity	Systolic longitudinal rotation could really detect ANT cardiotoxicity. Cardiac diastolic and systolic dysfunction was detected after ANT.
Boyd 2017 [39]	Longitudinal cohort	Breast cancer	NR	140	52 ± 9	Arterial hypertension 22% Diabetes mellitus 6% Hyperlipidemia 26% Tobacco use 25%	Doxorubicin 419 ± 67 mg/m^2^ epirubicin therapeutic equivalent 450 ± 136 mg/m^2^	Baseline and within seven days post treatment	E/A, DT; pulmonary vein flow, e‘, A‘, E/E‘, LA volume, diastolic strain	Peak E velocity, E/A ratio, pulmonary vein diastolic velocity, E’ velocity, and early diastolic strain rate reduction	Percentage change in early diastolic strain rate and E velocity predicted >11% reduction in systolic GLS.
Serrano 2015 [41]	Longitudinal cohort	Breast cancer	NR	85	49.7 ± 9	Arterial hypertension 22.4% Diabetes mellitus 7.1% Hyperlipidemia 10.6% Tobacco use 35.3%	Anthracycline 242 ± 4.5 mg/m^2^	Baseline, before last dose of acycline chemotherapy, 3 months, 9 months	E/A, DT, IVRT, e‘, E/e‘, color M-Mode propgation velocity	significant decrease in E’ at the septal and lateral mitral annuli and E/A, an increase in E/E’ratio, DT, IVRT.	Age and BMI were independent predictors of anthracycline-related diastolic dysfunction.
Florescu 2014 [29]	Longitudinal cohort	Breast cancer	0%	40	51 ± 8	Arterial hypertension 0% Diabetes mellitus 0% Hyperlipidemia 14% Tobacco use 28%	Epirubicin 268 ± 22 mg/m^2^	Baseline, after the third and last cycle anthracycline	E/A, e‘, E/e‘, color M-Mode propgation velocity, strain and strain rate by speckle tracking, rotation parameter	Reduction in E/A, and flow propagatin velocity, longitudinal strain rate, and untwist rate	Decreases in LV longitudinal strain and peak myocardial Velocities in systole after the third cycle of epirubicin predicted cardiotoxicity occurring after the sixth cycle.
Elitok 2014 [28]	Longitudinal cohort, randomised	Breast cancer	NR	40 without carvedilol	52.9 ± 11.2	CV risk factors were exclusion criteria	Doxorubicin 523.3 mg/m^2^	Baseline, after 6 months	E, A, E/A, IVRT	No significant difference	Decrease in LV basal septal and basal lateral peak systolic strain in the control group compared to the carvedilol group.
Akpek 2014 [30]	Randomised placebo controlled double-blind	Breast cancer	15%	40 without spironolactone	51 ± 10	Arterial hypertension was an exclusion criteria; other CV risk factors—NR	Total adriamycin dose (mg) 394.2 ± 71.9; total epirubicin dose (mg) 726.6 ± 120.5	Baseline and three week after the end of antracycline-based chemotherapy	E, A, E/A, DT, lateral e‘, E/e’	Reduction in lateral e‘	Significant deterioration of diastolic dysfunction grade was seen in the control group.
Stoodley 2013 [27]	Longitudinal cohort	Breast cancer	NR	52	49 ± 9	Arterial hypertension 4% Diabetes mellitus 4% Hyperlipidemia 12% Tobacco use 26%	Doxorubicin 236 ± 33 mg/m^2^ Epirubicin 408 ± 110 mg/m^2^	Baseline and 1 week post therapy	E, A, E/A, DT, LA volume index, pulmonary vein, e’, A’ velocities, diastolic strain by speckle tracking	A velocity, E/A reduced, early diastolic strain rate reduced	Reduced baseline systolic strain was found to be predictive of lower postchemotherapy E’.
Sawaya 2011 [26]	Longitudinal cohort	Breast cancer	NR	43	49 ± 10	Arterial hypertension 29% Diabetes mellitus 6% Hyperlipidemia 23% Tobacco use 15%	Doxorubicin 240 mg/m^2^; Epirubicin 300 mg/m^2^	Baseline, 3 months, 6 months after initiation of tratement	E, A, E/A, e‘, A’, E/e‘, LA volume	Reduction in e’	Decreases in longitudinal strain and radial strain and elevated high sensitive troponin at 3 months were predictive of patients who developed cardiotoxicity at 6 months.
Appel 2011 [25]	Longitudinal cohort	Breast cancer	NR	80	52 ± 9	Arterial hypertension 23.8% Diabetes mellitus 2.5% Hyperlipidemia NR Tobacco use 41.3%	Epirubicin 273.7 ± 46.6 mg/m^2^;	Baseline, after third cycle of Chemo	E, A, E/A, DT, Vp, e’, A’, E/e’, E/VP, E/A’	Reduction in E/A ratio	In contrast to several previous studies using tissue Doppler and conventional echocardiography, this study did not document changes in heart function with low-dose epirubicin.
Nagy 2008 [34]	Longitudinal cohort	Breast cancer	NR	40	49 ± 10	CV risk factors were exclusion criteria	Doxorubicin was 240 mg/m^2^, and the cumulative dose of epirubicin was 360 mg/m^2^	Baseline, 3, 6 months, 1 and 2 years	E, A, E/A, DT, e’, E/e’, IVRT, pulmonary veins velocities	Reduction in E/A, e’, increase in E/e’	Tissue Doppler imaging was a more precise and useful examination method than the traditional ones (E/A ratio or deceleration time) to demonstrate isolated diastolic dysfunction.

CV = cardiovascular; E = Mitral E-wave filling velocity; A = Mitral A-wave filling velocity; E/A = Mitral E-wave filling velocity/mitral A-wave filling velocity; e’ = Peak early diastolic mitral annular velocity; E/e’ = Mitral E-wave filling velocity/peak early diastolic mitral annular velocity; LA = left atrial; TR = tricuspid valve regurgitation; IVRT = isovolumetric ventricular relaxation time; DT = E-wave deceleration time; Vp = propagation velocity of the E wave; A’ = Peak late diastolic mitral annular velocity.

## Supplementary Materials

The following are available online at https://www.mdpi.com/article/10.3390/jcm10173890/s1, Figure S1: PRIMA flowchart for study selection. Figure S2. Funnel plot of standard error by standard difference in means to assess the risk of bias for the analysis of E/A changes before and after anthracycline-based chemotherapy. Figure S3. Funnel plot of standard error by standard difference in means to assess the risk of bias for the analysis of E/E’ changes before and after anthracycline-based chemotherapy. Figure S4. Funnel plot of standard error by standard difference in means to assess the risk of bias for the analysis of LVEF changes before and after anthracycline-based chemotherapy.
