# Supplementary material for: Left Ventricular Diastolic Function Following Anthracycline-Based Chemotherapy in Patients with Breast Cancer without Previous Cardiac Disease—A Meta-Analysis
Source: J Clin Med. 2021 Aug 29;10(17):3890. doi: 10.3390/jcm10173890 (PMC8432074; doi:10.3390/jcm10173890)

Left ventricular diastolic function following anthracycline-based chemotherapy in patients with breast cancer without previous cardiac disease – a meta-analysis

Raluca I Mincu, PhD[1]; Lena Lampe[1]; Amir A. Mahabadi, MD[1], Rainer Kimmig, MD[2], Tienush Rassaf, MD[1]; Matthias Totzeck, MD[1*]

[1]Department of Cardiology and Vascular Medicine, West German Heart and Vascular Center, University Hospital Essen, Hufelandstraße 55, 45147 Essen, Germany

[2]Clinic for Obstetrics and Gynecology, Essen University Hospital, Essen, Germany

Supplementary material

[#]Corresponding author:

Dr. Matthias Totzeck

University Hospital Essen

West German Heart and Vascular Center

Department for Cardiology and Vascular Medicine

Hufelandstr. 55, 45147 Essen, Germany

Tel.: +49 201 723 84805

Fax: +49 201 723 5401

E-mail: Matthias.Totzeck@uk-essen.de

Figure S1.

PRIMA flowchart for study selection

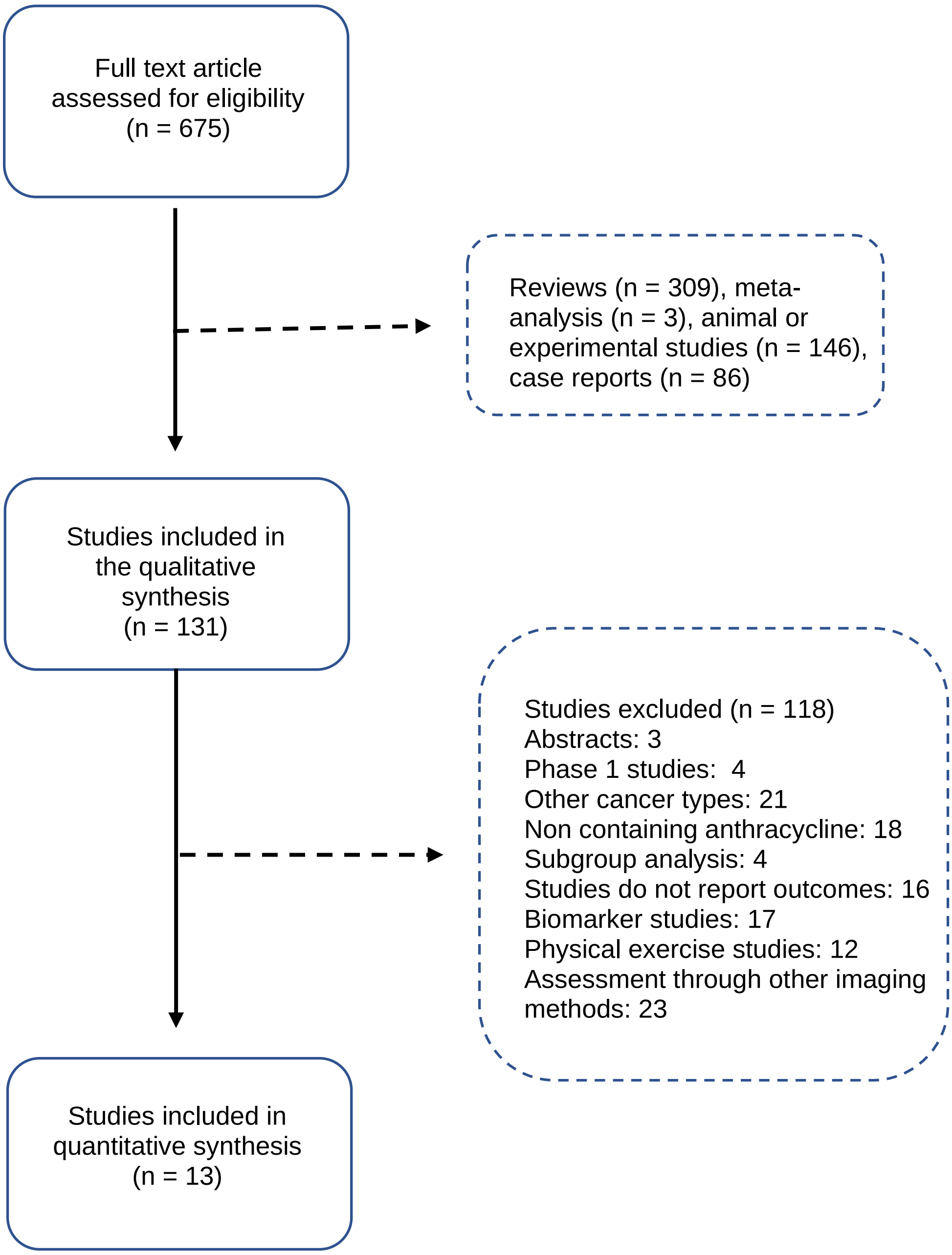

Figure S2. Funnel plot of standard error by standard difference in means to assess the risk of bias for the analysis of E/A changes before and after anthracycline-based chemotherapy.

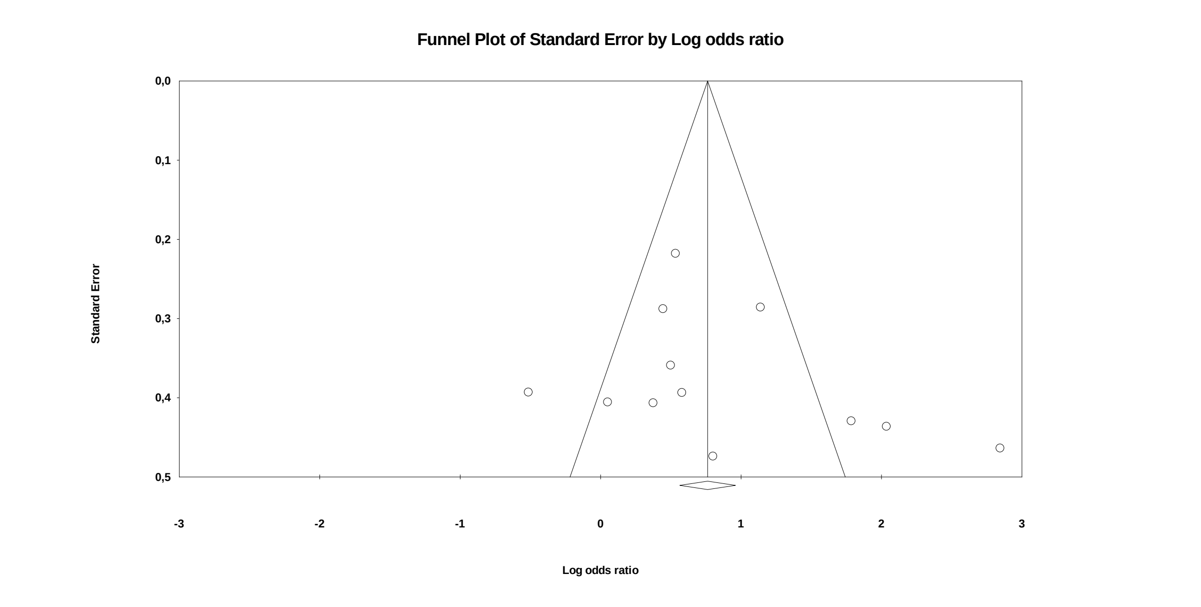

Figure S3. Funnel plot of standard error by standard difference in means to assess the risk of bias for the analysis of E/e’ changes before and after anthracycline-based chemotherapy.

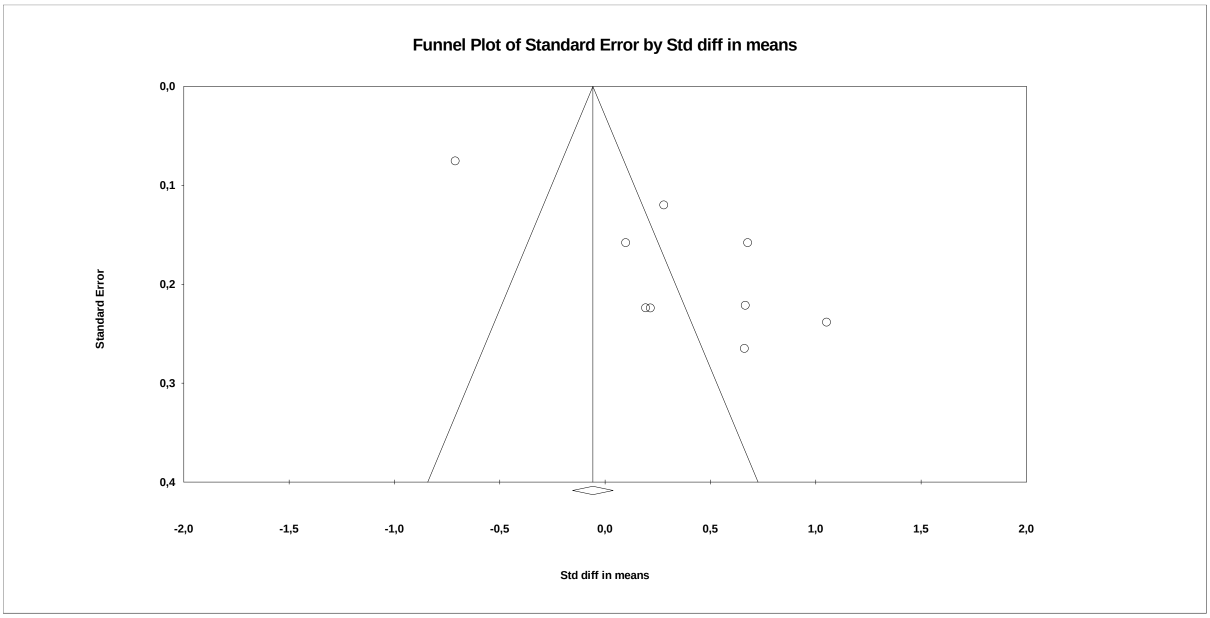

Figure S4. Funnel plot of standard error by standard difference in means to assess the risk of bias for the analysis of LVEF changes before and after anthracycline-based chemotherapy.

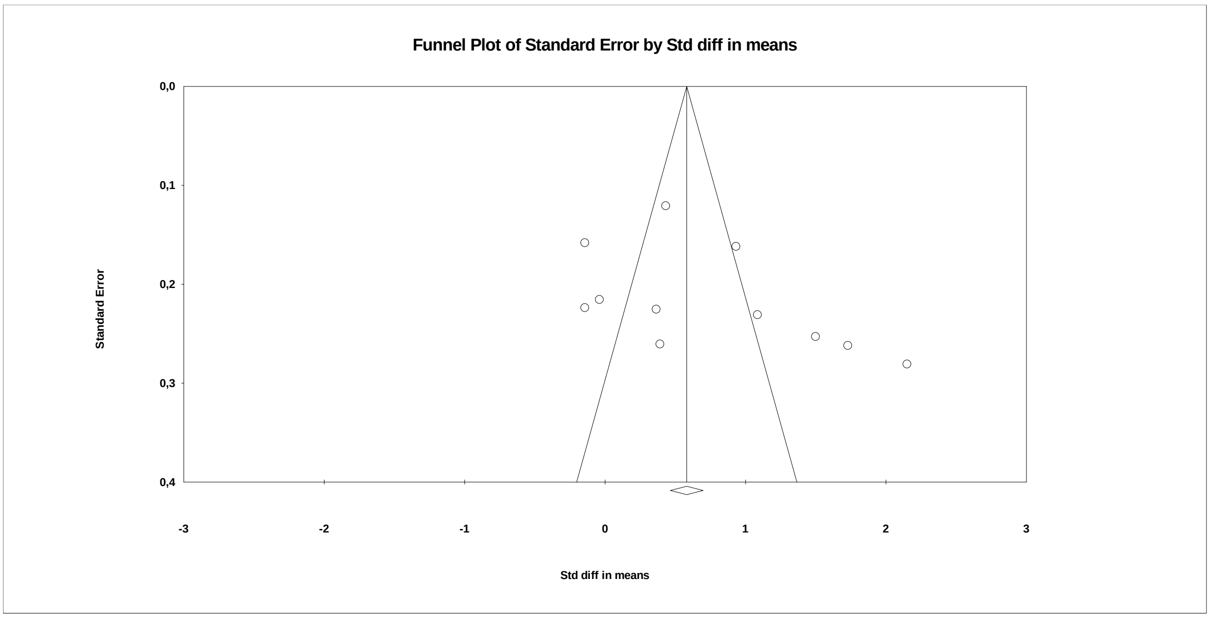

Supplement: Supplementary file 1 [file jcm-10-03890-s001.zip › jcm-1354044-supplementary.pdf]
